# Investigating the potential impact of dose banding for systemic anti-cancer therapy in the paediatric setting based on pharmacokinetic evidence

**DOI:** 10.1016/j.ejca.2017.11.029

**Published:** 2018-03

**Authors:** Melanie White-Koning, Caroline Osborne, Angelo Paci, Alan V. Boddy, Etienne Chatelut, Gareth J. Veal

**Affiliations:** a CRCT (Cancer Research Centre of Toulouse), Université de Toulouse, Inserm UMR 1037, Université Paul Sabatier, 31059 Toulouse Cedex 9, France; b Pharmacy Department, Alder Hey Children's NHS Foundation Trust, Liverpool L12 2AP, UK; c UMR CNRS 8203, Institut Gustave Roussy, 94805 Villejuif Cedex, France; d Faculty of Pharmacy, University of Sydney, Sydney, NSW 2006, Australia; e Institut Claudius Regaud, Institut Universitaire Du Cancer Toulouse-Oncopole, 31059 Toulouse Cedex 9, France; f Northern Institute for Cancer Research, Newcastle University, Newcastle Upon Tyne NE2 4HH, UK

**Keywords:** Dose banding, Paediatrics, Oncology, Dosing regimen, Pharmacokinetics

## Abstract

**Background:**

To make systemic anti-cancer therapy (SACT) preparation more practicable, dose-banding approaches are currently being introduced in many clinical centres. The present study aimed to determine the potential impact of using recently developed National Health Service in England (NHSE) dose-banding tables in a paediatric setting.

**Methods:**

Using pharmacokinetic parameters obtained from 385 drug administrations in 352 children aged from 1 month to 18 years, treated with five drugs (dactinomycin, busulfan, carboplatin, cyclophosphamide and etoposide), individual exposures (area under the plasma drug concentration versus time curve; AUC) obtained using doses rounded according to the published NHSE tables were calculated and compared with those obtained by standard dose calculation methods.

**Results:**

For all five drugs, the relative variation between the NHSE dose and the recommended dose (RecDose) (standard individually calculated dose) was between −6% and +5% as expected. In terms of AUC, there was no statistically significant difference in precision between exposures obtained by the RecDose and those obtained with dose banding (absolute value of relative difference 15–34%).

**Conclusion:**

Based on pharmacokinetic data for these five drugs, the results generated support the implementation of NHSE dose-banding tables. Indeed, inter-patient variability in drug clearance and exposure far outweighs the impact of relatively small drug dose changes associated with dose banding.

## Highlights

• The potential impact of using dose-banding tables in the setting of childhood cancer has been investigated.
• In 352 children receiving five anticancer drugs no difference was found in exposures using dose banding or recommended doses.
• Results generated support the implementation of National Health Service in England dose-banding tables.

## 1 Introduction

Drug dosing in oncology has historically been based on the body surface area (BSA) of the patient being treated [1]. According to the theory that larger patients have a higher elimination capacity, it is assumed that these patients need to be given higher doses than smaller patients to achieve comparable drug concentrations. For many drugs, plasma drug exposure (i.e. area under the plasma drug concentration versus time curve; AUC) is related to both toxicity and efficacy [2]. However, there is little or no direct correlation between BSA and AUC for most cytotoxic drugs, especially in adults [3]. It is arguably, therefore, somewhat surprising that the majority of anticancer drugs are still dosed based on an absolute calculation from BSA. Dose banding has recently been proposed to optimise chemotherapy preparations [4], [5], with ranges (or bands) of BSA, and corresponding midpoints of each band being predefined. The individual dose for a particular patient is calculated according to a single BSA value per band, usually the midpoint of the band in which the actual BSA of the patient lies. In a recent retrospective study, there was no significant difference in precision in reaching the target AUC for the AUC obtained by either dose banding or strict BSA-based dosing for 1012 adult patients treated with one of six anticancer drugs [6].

Many hospitals in England treating adult patients have now adopted a system of dose banding for systemic anti-cancer treatment (SACT), developed by NHS England's Medicine Optimisation and Chemotherapy Clinical Reference Groups [7]. In the National Health Service in England (NHSE) dose-banding system, calculated drug doses are grouped and rounded to a set of predefined doses. Each series of consecutive dose(s) is called a ‘band’, with the dose to which they are rounded towards being the ‘banded dose’. The NHSE bands have a maximum of 6% variance from the actual dose calculated, are defined by ‘measurable’ drug volume rather than a dose in milligrammes, and volumes consistent with normal vial sizes have been used to minimise waste where possible. Thanks to this system, chemotherapy provision can be rationalised and drugs with sufficient long-term stability can be prepared in advance of treatment. For doses that fall within commonly used dose bands, this can help rationalise chemotherapy service provision by enabling production, within a licenced hospital aseptic unit, or procurement from external compounding units, of standardised ready-to-use products. For less common dose bands, individualised dose preparation will still be required. The main advantages of this dose-banding approach include reduced patient waiting times and improved capacity planning of pharmacy production. Additional benefits include a reduced potential for medication errors, reduced drug wastage and prospective quality control of preparations. As recommended by the NHSE Clinical Reference Group, the national dose-banding tables are to be used by Hospital Trust Pharmacy Teams to ensure a standard approach to dose banding of chemotherapy across all hospitals. The initiative is initially focused on a relatively small number of commonly used drugs and is anticipated to help the NHSE to achieve improved values through the ability to purchase standard off-the-shelf products.

Although this approach has been demonstrated to be viable in adults, in children, the issue of chemotherapy dosing is rendered even more complex by developmental changes in organ function and by the ontogeny of drug metabolism and renal excretion, in addition to other sources of variability which also exist in adults, such as pharmacogenetic differences in drug disposition [8], [9]. Also, as the correlation between clearance and BSA or weight is better in children than in adults, it is important to conduct specific analyses on the acceptability of dose banding in the paediatric setting. Furthermore, protocol chemotherapy doses in paediatrics are often made on pragmatic empirical grounds, rather than on a sound pharmacological rationale, leading to the utilisation of diverse regimens, some based on BSA and others based on body weight. Of particular concern are the conversion rules from BSA-based drug dose regimens to weight-based dose regimens, as applied to the treatment of children under a certain age (e.g. less than 12 months) or under a certain weight (e.g. less than 10 or 12 kg) at seemingly arbitrary boundaries [9]. Thus, chemotherapy-dosing approaches designated for infants and young children in particular may lead to considerable inter-individual variability in drug exposure. This has recently been highlighted for the widely used anticancer drug carboplatin, with TDM approaches recommended over the variable BSA- or body weight–based dosing regimens previously employed [10].

The aim of the present study was to assess whether the dose-banding tables developed by the NHSE can be safely used in paediatric patients, according to pharmacokinetic criteria determined from previously published clinical trials. The individual exposures (AUC values) obtained using doses banded according to the NHSE tables (or banded using the same calculation method) were calculated and compared with those obtained with doses calculated according to standard methods for five commonly used anticancer drugs (dactinomycin, busulfan, carboplatin, cyclophosphamide and etoposide) administered to a total of 352 children and for which pharmacokinetic data were available from previously published paediatric studies.

## 2 Materials and methods

### 2.1 Patient eligibility and treatment

Our study included data from 352 children between the ages of 1 month and 17.7 years treated with at least one of the following five drugs: dactinomycin (n = 122), busulfan (oral administration: n = 25, intravenous (IV) administration: n = 58), carboplatin (n = 69), cyclophosphamide (n = 82) and etoposide (n = 29). All the children were included in clinical research studies, the details and conclusions of which have been published previously in all but one case [11], [12], [13], [14], [15], [16]. Exact doses administered and basic descriptive variables for all the children were available from each of these studies.

Unless otherwise stated, in all of the studies, the pharmacokinetic parameters (including individual drug clearance values) were derived from individual plasma concentration versus time profiles by fitting a population pharmacokinetic model to the data using NONMEM, version 6 or 7.2 [17].

### 2.2 Dactinomycin

Of the 122 patients included in the present work, six patients were part of a pilot study published in 2005 [11] (Act D PK 1) and the remaining 116 patients were from a follow-up study published in 2014 [12] (Act D PK 2).

In Act D PK 1, dactinomycin (Act D) was administered as an IV bolus at doses between 0.7 and 1.5 mg/m^2^ as part of the standard chemotherapy regimen that each patient was receiving. Blood samples for measurement were obtained from a central line before administration and at 15 and 30 min and 1, 2, 4, 6 and 24 h after administration. Plasma concentrations of dactinomycin were measured using a validated liquid chromatography-mass spectrometry (LC/MS) assay. Pharmacokinetic modelling and parameter estimation were carried out using WinNonlin Professional, version 3.1 software (Pharsight Corp, Mountain View, CA).

In Act D PK 2, dactinomycin was administered intravenously (1–5 min) at doses between 0.4 and 1.6 mg/m^2^, with maximum dose capped at 2 mg for larger children. The dose of dactinomycin administered was adjusted for infants aged <1 year, or weighing <10 kg in body weight, with protocol doses of 0.02–0.05 mg/kg. Blood samples for measurement were collected from a central venous line before administration and at 5, 15 and 30 min and 2, 4, 8, 24 and 26 h after administration. Plasma concentrations of dactinomycin were obtained using a modified LC/MS assay, and population pharmacokinetic modelling was carried out as described in Ref. [12].

### 2.3 Busulfan

Busulfan data were obtained from patients being treated on the High Risk NeuroBLastoma trial-1 (HR-NBL-1) study [14], with busulfan administered four times daily for 4 days. Oral busulfan (25 patients) was administered at a dose of 30 mg/m^2^ (1.45 mg/kg) for children <12 kg and at a dose level of 37.5 mg/m^2^ (1.55 mg/kg) for patients >12 kg (as these patients were in the original study, we decided to include them in the present analysis using the same banding method as the parenteral form). Intravenous busulfan (58 patients) was administered over 2 h at five fixed dose levels from 0.8 to 1.2 mg/kg, according to body weight and without dose adaptation. Blood samples were obtained before administration and at 2, 4 and 6 h after the start of administration on day 1 of treatment. An additional sample was obtained before the start of administration for doses 5, 9 and 13. Busulfan analysis was carried out using gas chromatography-mass spectrometry [18].

### 2.4 Carboplatin

Of the 69 patients receiving carboplatin, 28 were from the MMT 98 study of soft-tissue sarcoma patients [19], 19 patients were from the European Infant Neuroblastoma Study (INES) study of children aged <1 year at diagnosis and weighing <12 kg at treatment [16], and the remaining 22 patients were from the INF PK/PG study (REC 06/MRE04/46; CTA: 17136/0245/001; European Clinical Trials Database (EUDRACT): 2006-002845-36), an unpublished study investigating the pharmacokinetics of several anticancer drugs in children aged 0–2 years (doses described in detail in Table 1).

**Table 1:** Patient and drug characteristics for each drug group.

Drug (Total number of drug doses)	Studies	Dosing regimen	Number of patients	Age (years)		Weight (kg)	
				Median	(min–max)	Median	(min–max)
**Actinomycin D**	**Act D PK 2 (Hill *et al.*, 2014)**	0.025 mg/kg	2	4.6	(0.4–9)	15.0	(9–21)
**(N** = **122)**	n = 116	0.030 mg/kg	22	1.6	(0.8–15)	10.9	(7–75)
		0.045 mg/kg	34	3.9	(0.3–17)	16.2	(5–50)
		0.05 mg/kg	4	1	(0.9–1.2)	8.8	(8–10)
		0.75 mg/m^2^	17	8.1	(2–17)	25.4	(13–77)
		1.5 mg/m^2^	36	5.5	(2–14)	19.0	(11–63)
		*Unknown*	1	16	–	59.5	–
	**Act D PK 1 (Veal *et al.*, 2005)**	0.030 mg/kg	1	15.5	–	63.0	–
	n = 6	0.045 mg/kg	1	3.2	–	16.3	–
		0.75 mg/m^2^	1	17.7	–	62.5	–
		1.5 mg/m^2^	3	6.3	(3–15)	30.5	(10–33)
**Busulfan**	**HR-NBL-1 (Veal *et al.*, 2012)**						
**(N** = **83)**	Oral busulfan: n = 25	30 mg/m^2^	8	2.1	(2–3)	11.1	(10–12)
		37.5 mg/m^2^	17	3.9	(3–8)	16.6	(12–23)
	IV busulfan: n = 58 (+20^a^)	1.0 mg/kg	4	1.0	(1–9)	8.5	(8–25)
		1.1 mg/kg	14	4.5	(2–7)	17.8	(13–21)
		1.2 mg/kg	39	3.0	(1–6)	13.9	(10–20)
		1.3 mg/kg	1	5.5	–	14.3	–
**Carboplatin**	**INES (Veal *et al.*, 2010)**	3.75 mg/kg	1	0.2	–	4.8	–
**(N** = **69)**	n = 19	5 mg/kg	1	0.5	–	7.4	–
		6.6 mg/kg	17	0.8	(0.2–1)	8.7	(5–11)
	**INF PK/PG**^a^	6.6 mg/kg	4	0.6	(0.5–1)	8.6	(7–11)
	n = 21	300 mg/m^2^	1	1.9	–	11.8	–
		500 mg/m^2^	2	1.9	(1.8–2)	12.5	(12–13)
		550 mg/m^2^	3	1.5	(0.6–2)	8.7	(8–12)
		*Unknown*	11	0.7	(0.1–2)	8.8	(4–12)
	**MMT 98 (Veal *et al.*, 2007)**						
	n = 28(+1^b^)	GFR-based	29	12.0	(1–17.5)	38.5	(10–88)
**Cyclophosphamide**	**INF PK/PG**^a^	5 mg/kg	2	0.4	(0.4–0.4)	8.3	(7–9)
**(N** = **82)**	n = 21	10 mg/kg	3	1.7	(0.8–2)	11.8	(9–13)
		36 mg/kg	2	0.5	(0.3–1)	7.1	(6–9)
		45 mg/kg	4	1.9	(1–2)	12.0	(11–16)
		750 mg/m^2^	3	0.7	(0.7–1)	8.7	(7–10)
		330 mg/m^2^	1	1.9	–	11.8	–
		1500 mg/m^2^	2	1.9	(1.8–1.9)	12.9	(10–16)
		*Unknown*	4	0.9	(0.8–1)	9.0	(8–11)
	**Cyclo NHL (Veal *et al*., 2016)**						
	n = 47	250 mg/m^2^	47	11	(3–17)	36.2	(13–82)
	**MMT 98 (Chinnaswamy, 2011)**						
	n = 14	2 g/m^2^	14	13.0	(5–17.5)	48.3	(22–85)
**Etoposide**	**INES (Veal *et al*., 2010)**						
**(N** = **29)**	n = 11	5 mg/kg	11	0.9	(0.2–1)	8.8	(5–10)
	**INF PK/PG**^a^	3.3 mg/kg	2	0.3	(0.1–0.4)	4.6	(3–6)
	n = 18	5 mg/kg	3	0.5	(0.4–0.7)	7.4	(7–8)
		7.5 mg/kg	1	1.5	–	10.6	–
		12 mg/kg	2	0.8	(0.8–0.8)	8.6	(8–9)
		75 mg/m^2^	1	1.9	–	11.8	–
		100 mg/m^2^	1	1.8	–	10.8	–
		120 mg/m^2^	3	1.3	(1–2)	11.0	(11–12)
		150 mg/m^2^	3	1.3	(1–2)	12.0	(5–15)
		*Unknown*	2	1.2	(0.9–2)	9.6	(7–12)

IV, intravenous.

Note: some patients were treated with more than one drug so their data appears in more than one drug group.

a These patients are from unpublished studies.

b Patient from INF PK/PG study.

In the MMT 98 study, 28 patients were treated with high-dose carboplatin administered as a 1 h IV infusion, with the initial dose based on renal function, to achieve cumulative target AUC values of 20 mg/mL min over a 5-day treatment period. Dose adjustment was carried out based on observed individual daily AUC values to obtain the defined target exposure. Blood samples for pharmacokinetic analysis were obtained from a central line before infusion and at 30 min, 60 min and 120 min after the start of infusion.

In the INES study, carboplatin (6.6 mg/kg/day; 1 h IV infusion) and etoposide were co-administered to the 19 patients on each of 3 days of treatment. Blood samples for measurement of carboplatin concentrations were taken before infusion and at 30 min, 1 h and 2 h after the start of administration.

In both of these studies, the samples were centrifuged to obtain plasma ultrafiltrate for the determination of free carboplatin levels. Platinum pharmacokinetic analyses were carried out by flameless atomic absorption spectrophotometry. Unbound platinum levels were determined in plasma ultrafiltrate samples as described in Ref. [20].

### 2.5 Cyclophosphamide

Of the 82 patients receiving cyclophosphamide, 47 patients were from the Cyclo NHL study of B-cell non-Hodgkin lymphoma patients [13]; Veal *et al.*, 2016, 14 were from the MMT 98 study [15], and the remaining 21 patients were from the unpublished Infant PharmacoKinetics/PharmacoGenetics (INF PK/PG) study involving children aged 0–2 years (doses described in detail in Table 1).

In the Cyclo NHL study, cyclophosphamide (250 mg/m^2^) was administered as a 15 min IV infusion twice daily on days 2, 3 and 4 of treatment as part of the COPADM regimen (i.e. with vincristine, prednisolone, doxorubicin, methotrexate, folinic acid and intrathecal methotrexate/hydrocortisone). Blood samples were obtained before administration of the first dose of cyclophosphamide on day 2, at the end of infusion and at 1, 2, 4, 6 and 12 h after the start of infusion. Concentrations of cyclophosphamide were measured using a validated LC/MS method [15].

### 2.6 Etoposide

In the INES study, etoposide (5 mg/kg/day; 2 h IV infusion) and carboplatin were co-administered to the patients on each of 3 days of treatment. Blood samples for the measurement of etoposide concentrations were taken before infusion and at 1 h, 2 h and 4 h after the start of administration from 11 patients. Etoposide levels were determined using an API 2000 LC/MS/MS after extraction from plasma samples. The remaining 18 patients treated with etoposide were from the unpublished INF PK/PG study (doses described in detail in Table 1).

### 2.7 Calculation of drug doses using the NHSE dose-banding tables

For each drug and dosing regimen (except Glomerular Filtration Rate (GFR)-based carboplatin), the recommended dose (RecDose) was calculated as the dosing regimen stated in the protocol adapted to child weight [RecDose = dosing regimen (mg/kg) × weight (kg)] or child BSA [RecDose = dosing regimen (mg/m^2^) × BSA (m^2^)] as defined in the treatment protocol, with BSA calculated using body weight/BSA conversion tables provided by the Children's Cancer and Leukaemia Group Chemotherapy Standardisation Group [21]. For carboplatin administered according to patient GFR, the target AUC was 4 mg/mL min, and the RecDose was calculated according to the formula used in MMT 98: RecDose (mg) = 4 (mg/mL min) × [GFR (mL/min) + 15 (ml/min/m^2^) × BSA (m^2^)].

For each patient, the dose which would have been administered using the NHSE rounding tables [7] was obtained by taking the RecDose and finding the corresponding band dose given in the appropriate table (6 mg/mL table for busulfan, 10 mg/mL table for carboplatin and 20 mg/mL table for cyclophosphamide and etoposide). Once reconstituted, dactinomycin has a concentration of 0.5 mg/mL; however, there is no NHSE dose-banding table published yet for this concentration. In addition, some of the doses administered for busulfan were below those given in the published tables. In both these cases, we were able to use unpublished tables provided by the NHSE Chemotherapy Dose Standardisation Group which were banded according to the same calculation method (see tables in supplementary data). This rounded dose was then referred to as the NHSE dose.

### 2.8 Impact of dose banding on patient treatment

The relative difference between each dose calculation method and the recommended dose (RecDose) was calculated in the following way: Relative difference = ([DoseStudied–RecDose]/RecDose) × 100; where DoseStudied is the actual dose administered (ActualDose) or the NHSE dose. The absolute value of relative difference, which is = (|DoseStudied–RecDose|/RecDose) × 100, was also calculated. Mean and standard deviation for these quantities were given as well as minimum and maximum values for relative difference. As an indication of dose modification, the percentage of values where the absolute value of relative difference was greater than 5% was also given.

For each drug and each dosing regimen, the target AUC was calculated by dividing the RecDose by the mean value of observed clearance (CL) expressed in mL/min: target AUC = RecDose (mg)/mean observed CL (mL/min) (except for GFR-based carboplatin where target AUC = 4 mg/mL min as defined in the MMT 98 study protocol). For busulfan, as well as the theoretical individual target AUC calculated as described above, we also described percentage of AUC values falling within the ‘therapeutic window’ AUC of 900–1500 μM min (0.22–0.37 mg/mL min) which has been defined by TDM approaches for busulfan [22], [23]. For each patient, the AUC corresponding to the ActualDose was calculated as AUC_Actual = ActualDose/individual clearance and the AUC corresponding to the NHSE dose was calculated as AUC_NHSE = NHSE dose/individual clearance. The percentage error of AUC (π) corresponding to each dose was calculated: e.g. [(AUC_NHSE–target AUC)/target AUC] × 100. The absolute value of relative difference (|AUC_NHSE–target AUC|/target AUC) × 100 was also calculated. The precision (root mean square error, RMSE) corresponding to each dosing method was calculated in the following way: RMSE (%)=$\sqrt{\sum_{i=1}^{n}\left(\text{π}\right)²/\text{n}}$, where n is the number of patients in each drug group.

The potential impact of using NHSE dose banding was evaluated in three different ways. First, we calculated the relative difference and absolute value of the relative difference (described above) between the NHSE dose and the RecDose. Second, the absolute value of the relative difference in AUCs obtained using the NHSE dose and the RecDose was compared using the paired Student t-test or the Wilcoxon signed-rank test (depending on data normality). Finally, the paired Student t-test or Wilcoxon signed-rank test was used to compare the precision (RMSE) of the AUCs obtained using the NHSE dose to the precision of the AUC obtained with the RecDose.

## 3 Results

### 3.1 Patient characteristics and treatment

The study included 385 drug administrations from 352 children aged between 1 month and 17.7 years (median = 3.6 years), being treated for cancer with at least one of the following drugs: dactinomycin (122 drug administrations), busulfan (83), carboplatin (69), cyclophosphamide (82), and etoposide (29). Some children were treated with several drugs: 11 children from the INES study were treated with carboplatin and etoposide, 11 children from the MMT 98 study were treated with carboplatin and cyclophosphamide, and in the INF PK/PG study, the treatment combinations were carboplatin, cyclophosphamide and etoposide (1 patient), cyclophosphamide and etoposide (2 patients), carboplatin and cyclophosphamide (2 patients) and carboplatin and etoposide (5 patients). These children were treated for a wide range of tumours including neuroblastoma (117 children), B-cell non-Hodgkin's lymphoma (47), Wilms tumour (45), rhabdomyosarcoma (31), other soft-tissue sarcomas (32) and Ewing's sarcoma (26) (see Table 2 for details of all tumour types). As can be seen in Table 1, even for patients treated within the same study, there was a wide variety of dosing regimens used for each drug. Some dosages were weight-based, with others based on BSA or GFR (for carboplatin). In terms of age, the etoposide group and the non-GFR-based carboplatin group were all younger than 2 years, and the children in the busulfan group were all younger than 8 years. In the other drug groups, the age range was wider, extending from infants to teenagers.

**Table 2:** Tumour types in each drug group.

Drug	Tumour	Frequency	Percent
Dactinomycin (n = 122)	Wilms	45	36.9
	Rhabdomyosarcoma	31	24.6
	Ewing's sarcoma	25	20.5
	Alveolar rhabdomyosarcoma	7	5.7
	Embryonal rhabdomyosarcoma	6	4.9
	Rhabdomyosarcoma of prostate	1	0.8
	Embryonal sarcoma of liver	1	0.8
	Malignant mesenchymal tumour	1	0.8
	Metastatic pancreatic neuroendocrine tumour	1	0.8
	Metastatic non-rhabdomyosarcoma	1	0.8
	Nephroblastoma	1	0.8
	Non-rhabdomyosarcoma	1	0.8
	Paraspinal undifferentiated sarcoma	1	0.8
	Pleuropulmonary blastoma	1	0.8
	Soft-tissue sarcoma	1	0.8
Busulfan (n = 83)	Neuroblastoma	83	100
Carboplatin (n = 69)	Soft-tissue sarcoma	28	40.6
	Neuroblastoma	26	37.7
	Retinoblastoma	4	5.8
	Bilateral retinoblastoma	2	2.9
	Ependymoma	1	1.5
	Germ-cell tumour	1	1.5
	Optic chiasm Glioma	1	1.5
	PNET supratentorial	1	1.5
	Rhabdoid	1	1.5
	Rhabdoid – kidney	1	1.5
	Rhabdoid sarcoma	1	1.5
	Vaginal yolk sac tumour	1	1.5
	Visual pathway glioma	1	1.5
Cyclophosphamide (n = 82)	B-cell non-Hodgkin lymphoma	47	57.3
	Soft-tissue sarcoma	14	17.1
	Acute lymphoblastic leukaemia	8	9.8
	Neuroblastoma	6	7.3
	Astrocytoma	1	1.2
	Ependymoma	1	1.2
	Optic chiasm glioma	1	1.2
	PNET supratentorial	1	1.2
	Posterior fossa ATRT	1	1.2
	Rhabdoid – kidney	1	1.2
	Rhabdomyosarcoma	1	1.2
Etoposide (n = 29)	Neuroblastoma	18	62.1
	Retinoblastoma	4	13.8
	Acute lymphoblastic leukaemia	1	3.5
	Astrocytoma	1	3.5
	Ewing's sarcoma	1	3.5
	Germ-cell tumour	1	3.5
	Rhabdoid – kidney	1	3.5
	Teratoma	1	3.5
	Yolk sac tumour	1	3.5

Note: some patients are included in more than one treatment group.

### 3.2 Pharmacokinetics and dosing

**Fig. 1:**
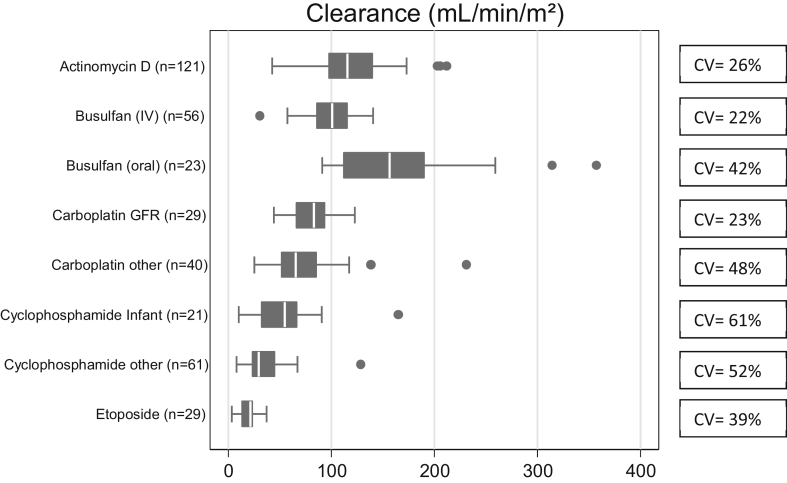
Individual mean clearance values (mL/min/m^2^) according to drug type with coefficient of variation (CV) as measure of variability.

As can be seen in Fig. 1, the inter-individual variability in individual mean clearance values ranged from 22% (coefficient of variation) for busulfan (IV) to 61% for the infant cyclophosphamide group. With the exception of busulfan (IV) and GFR-based carboplatin, all other drug groups exhibited inter-individual variabilities greater than 25%.

**Fig. 2:**
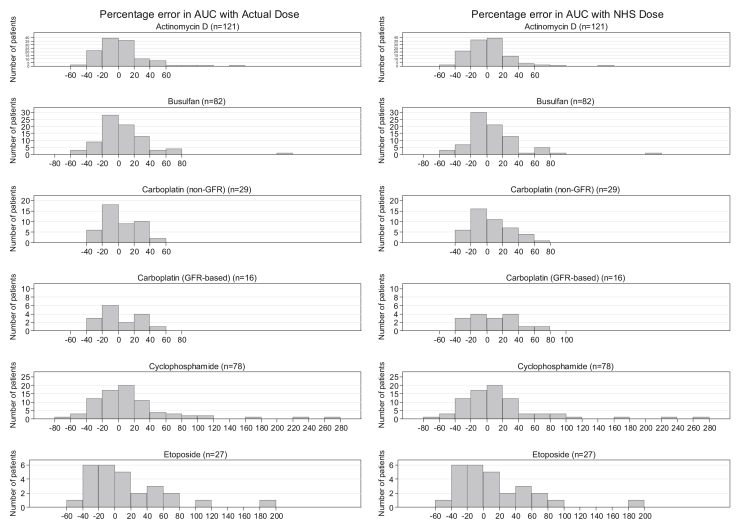
Frequency of percentage errors between individual area under the curve (AUC) and target AUC using standard dosing methods (Actual dose) or NHSE dose-banding (NHS dose) for dactinomycin, busulfan, carboplatin, cyclophosphamide and etoposide. NHSE, National Health Service England.

**Fig. 3:**
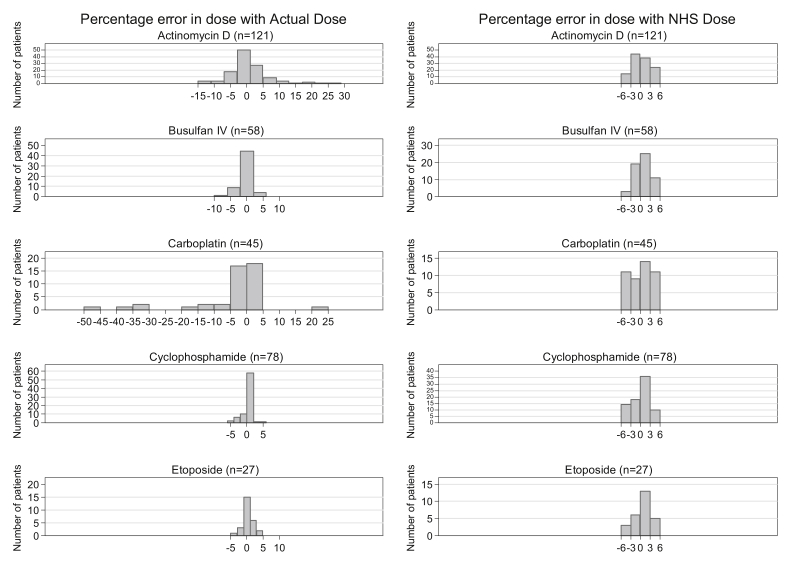
Frequency of percentage errors observed in the actual doses administered (Actual dose) as compared with the proposed NHSE dose-banding doses (NHS dose) for dactinomycin, busulfan, carboplatin, cyclophosphamide and etoposide. NHSE, National Health Service England.

Over all five drugs studied, the relative variation in dose between the ActualDose and the RecDose according to the stipulated dosing regimen ranged between −43% and +26% (Table 3). In contrast, the difference was between −6% and +5% for the NHSE-rounded dose relative to the RecDose (Table 3), which was to be expected as the NHSE tables were constructed for the variation to be less than 6%. These differences were small for busulfan, cyclophosphamide and etoposide (between −7% and +7%) but were much greater for carboplatin (GFR-based dosing between −43% and +1% and other dosing regimens between −32% and +3%) and dactinomycin (between −14% and +26%). These observed discrepancies from the stipulated dosing regimens may represent dose adjustments relating to dose capping, liver impairment, co-morbidities or in the case of carboplatin, cautionary approaches to initial dosing ahead of adaptive dosing. In terms of AUC and the capacity to attain the target AUC, there was no statistically significant difference in precision for any of the five drugs between the plasma exposure obtained with the standard dosing method (Actual dose) and that obtained with the NHSE dosing method, with RMSEs ranging from 21% (for carboplatin) to 57% (for cyclophosphamide) (Table 4). Fig. 2 shows the frequency of percentage errors between individual AUC and target AUC using standard dosing methods (Actual dose) or the NHSE dose-banding method for each drug, with very similar distributions except for carboplatin, where the distributions were more variable. For busulfan, the proportion of AUC values falling within the therapeutic window of 900–1500 μM min was comparable, whichever dosing method was used. Of relevance to the utility of NHSE dose banding, for patients treated with IV busulfan, 74% of their actual AUC values were within the therapeutic window, whereas 79% of the AUC values calculated with the NHSE dosing method were within the therapeutic window. Fig. 3 shows the frequency of percentage errors observed in the ActualDoses as compared with the proposed NHSE dose-banding doses (NHSE Dose) for all five drugs studied. Fig. 4SA and 4SB are given as supplementary data and show, for each of the five drugs under study, clearance (mL/min) versus body weight (kg) (Fig. 4SA) and BSA (m^2^) (Fig. 4SB).

**Table 3:** Percentage dose variation of actual dose administered and NHSE dose-banding method relative to recommended dose.

Drug	N	Actual dose				NHSE dose			
		Absolute value of relative difference		Relative difference		Absolute value of relative difference		Relative difference	
		Mean (SD)	≥5%^a^	Min	Max	Mean (SD)	≥5%^a^	Min	Max
**Dactinomycin**	121	4.0 (4.8)	26	−14	+26	2.3 (1.3)	1	−5	+5
**Busulfan**	83	1.6 (1.6)	5	−7	+7	1.9 (1.2)	1	−4	+5
Busulfan (oral)	25	1.9 (1.9)	8	−5	+7	1.9 (1.3)	4	−2	+5
Busulfan (IV)	58	1.5 (1.5)	3	−7	+4	1.9 (1.2)	0	−4	+4
**Carboplatin (other)**	29	2.1 (5.8)	0	−32	+3	2.6 (1.4)	0	−5	+4
**Carboplatin (GFR)**	16	3.3 (10.6)	6	−43	+1	3.3 (1.3)	0	−5	+4
**Cyclophosphamide**	78	0.7 (1.1)	0	−4	+5	2.3 (1.4)	4	−6	+5
**Etoposide**	27	1.1 (1.3)	0	−4	+4	2.0 (1.5)	0	−4	+5

$\text{Relative}\;\text{difference}=\frac{\left(\mathrm{DoseStudied}-\mathrm{RecDose}\right)}{\mathrm{RecDose}}\times 100$ where DoseStudied is Actual dose or NHSE dose.

$\text{Absolute}\;\text{value}\;\text{of}\;\text{relative}\;\text{difference}=\frac{\left|DoseStudied-RecDose\right|}{RecDose}\times 100$.

NSHE, National Health Service in England; SD, standard deviation.

a Percentage of values where absolute value of relative difference is greater than 5%.

**Table 4:** Differences in plasma exposure (AUC) using each dosing method (Actual dose and NHSE dose) compared with target AUC [recommended dose (mg)/mean CL (mL/min)].

Drug	N	AUC with Actual dose		AUC with NHSE dose	
		Absolute value of relative difference		Absolute value of relative difference	
		Mean (SD)	RMSE	Mean (SD)	RMSE
**Dactinomycin**	121	19.9 (21.2)	29.0	19.2 (19.5)	27.3
**Busulfan**	82	22.2 (26.8)	34.7	22.8 (27.2)	35.4
Busulfan (oral)	25	28.4 (20.2)	34.6	29.5 (20.8)	35.8
Busulfan (IV)	57	19.5 (29.0)	34.7	19.9 (29.3)	35.1
**Carboplatin (other)**	29	16.2 (14.1)	21.3	15.3 (14.4)	20.8
**Carboplatin (GFR)**	16	22.3 (15.2)	26.7	25.5 (16.4)	30.1
**Cyclophosphamide**	78	34.0 (45.5)	56.6	34.1 (45.5)	56.6
**Etoposide**	27	33.4 (39.3)	51.0	34.2 (38.9)	51.2

RMSE, root mean square error; NHSE, National Health Service England; SD, standard deviation

## 4 Discussion

Although BSA-based dosing has been criticised for many years now [24], because of the weak correlation between drug clearance (and thus conversely systemic exposure) and BSA, it remains widely used in both adults and children. In the paediatric setting, variations in inter-individual metabolism due to developmental changes, in addition to other sources of variability, can result in significant differences in drug exposure for children compared with adults treated at the same dose [25]. Pharmacokinetic studies have shown that children receiving the same dose scaled to BSA or body weight commonly exhibit large differences in systemic drug exposure, which in turn may be associated with sub-therapeutic drug concentrations or overexposure [26]. Another significant problem is the use of arbitrary dose reductions for infants, using either a percentage according to age (e.g. 50% dose reduction under 12 months) or body weight (10 kg or 12 kg thresholds are commonly used), based on fears of an increased susceptibility to toxicity but with little scientific justification [27]. The dearth of information concerning the pharmacokinetics of many anticancer drugs, particularly for infants below the age of 12 months, is linked to a lack of scientific rationale and standardisation in terms of dosing regimens. A recent report by Balis *et al.* (2017) from the Children's Oncology Group Chemotherapy Standardization Task Force, showed once again that BSA and body weight are inconsistently used across drugs and treatment protocols (sometimes even within protocols) to adjust doses for the wide range of body sizes encountered from birth to adulthood. In the 29 Children's Oncology Group protocols studied, 11 sets of criteria using age, weight, BSA or a combination of these parameters were used for dose modifications as well as eight dose modification methods.

This heterogeneity in approaches to dosing is apparent from the current analysis, with a variety of dosing regimens used for the five drugs even within the same clinical study or for the same tumour type (Table 1). Over and above these variations in intended or ‘recommended dose’ (i.e. the dosing regimen specified in the protocol), our analysis indicated that there was also a difference between the actual dose administered and the recommended dose. Although the absolute values of mean differences are relatively small (0.7–3.9%), some individual relative differences are much larger, with dactinomycin values ranging between −14% and +26% and carboplatin values down to −43% for GFR-based carboplatin and −32% for carboplatin based on other dosing regimens (Table 3). For example, the patient for whom the relative difference is −43% is an 11-year-old girl treated for soft-tissue sarcoma who should have received an initial dose of 526 mg of carboplatin based on GFR and the formula used to attain the target AUC (see Methods section), but the actual first dose she received was 300 mg. Interestingly, the dose was increased on subsequent days of treatment as therapeutic drug monitoring (TDM) was carried out on this patient to achieve the target AUC, highlighting the benefits of TDM approaches to treatment in a paediatric oncology setting [9], [27]. It should be noted that this type of clinical scenario, incorporating an initial lower dose followed by adaptive dosing, could be applied equally to both standard dosing and dose-banding approaches.

The absolute value of relative differences in plasma exposure between the target AUC (based on the recommended dose) and the actual AUC, ranged from 20% for dactinomycin and busulfan administered by IV to 34% for cyclophosphamide. These mean differences can result from differences in doses as mentioned above and inter-individual variability in terms of drug clearance (see Fig. 1).

In comparison, the doses which would have been administered based on the banded doses proposed in the NHSE tables had relative differences ranging from −6% to +5% for all five drugs examined (Table 3) as expected. Importantly, differences between target AUC values and AUC values which would have been obtained with NHSE dose banding were not statistically significantly different from the variability in plasma exposure already observed with current dosing practices (Table 4). Fig. 2 also shows that the distribution of percentage errors between individual AUC and target AUC values is comparable for all five drugs when using NHSE dose banding and standard dosing methods. For busulfan, the proportion of AUC values falling within the therapeutic window of 900–1500 μM min was unaffected by the dosing method used and was comparable to that found in the article initially reporting these data [14]. These results are in agreement with a previously published retrospective study including 1012 adult patients treated with one of six anticancer drugs, where the authors found no significant difference in precision in reaching the target AUC between dose-banding and BSA-based dosing [6].

The Chemotherapy Dose Standardisation initiative, developed by NHS England's Medicine Optimisation and Chemotherapy Clinical Reference Groups, has published dose-banding tables to be used for a number of drugs by Hospital Trust Pharmacy Teams to ensure a standard approach to dose banding of SACT across all Hospital Trusts [7]. The guiding principle for these tables was: ‘no target dose of traditional SACT is greater than ±6% of the precise calculated dose without specific prior agreement and no target dose of [monoclonal antibodies] used as a SACT is greater than ±10% of the precise calculated dose without specific prior agreement’. Also the dose bands used in these tables were calculated as a measurable drug volume rather than a dose in milligrammes, hence they can be applied to any drug, and volumes which closely match vial sizes have been used where possible to minimise waste.

For drugs with sufficient long-term stability, preparation of commonly banded doses can be carried out in advance, which can help rationalise chemotherapy service provision and reduce patient waiting times. Additional benefits include a reduced potential for medication errors, reduced drug wastage, prospective quality control of preparations and reduced workload for staff. These will of course also have a positive impact on the overall cost of chemotherapy for the health service, with the money saved potentially reinvested into research or other schemes to improve paediatric cancer care. Pharmacoeconomic studies comparing costs with and without the use of dose banding could be conducted to verify this. Many of these benefits are not transferable to the paediatric setting as the dose bands used will not be those commonly used in adults, and final drug volumes of ready-to-use products may be too high for young patients to tolerate, thus requiring individualised dose preparation. In addition, many children with cancer are treated on clinical trials (national or international) which may not currently allow dose banding. Nevertheless, for those centres treating adults, children and young people, benefits may be seen in the older children, particularly related to the use of standardised ready-to-use products.

In a recent effort to rationalise and simplify the array of anticancer drug-dosing methods used in the Children Oncology Group (COG) trials, the COG's Chemotherapy Standardization Task Force have developed dosing tables for infants and children with a BSA<0.6 m^2^ (which is reached at about 36 months of age) [9]. The tables are different for each drug and give doses for defined BSA bands and gradually transition stepwise from the dose based on body weight using the 30-Rule (dividing the BSA-based dose by 30) to BSA dosing. Based on data from 1718 infants and children treated on COG trials, a simple linear regression model was used to obtain dose values and then dose bands based on BSA intervals and deliverable drug concentrations and volumes. The authors emphasise that these tables are empirical, and it remains to be determined whether this infant dosing method provides more uniform exposure across patients and over the entire age range (birth to 36 months) by studying pharmacokinetic data. Based on pharmacokinetic data from 352 children, our study supports the implementation of dosing based on the NHSE dose-banding tables, at least for the five drugs investigated here (dactinomycin, busulfan, carboplatin, cyclophosphamide and etoposide). Further evaluation of the effect of using dose banding for other drugs would be needed to confirm these results and extend them to additional anticancer drugs.

## Conflict of interest statement

Caroline Osborne works as a cancer pharmacist for the NHSE North of England Specialised Commissioning Team (North-West Hub) under a service level agreement. All the other authors declare no potential conflicts of interest.

## Appendix A Supplementary data

**Fig. 4SA**: Clearance (mL/min) versus body surface area (m^2^) according to drug type.

**Fig. 4SB**: Clearance (mL/min) versus body weight (kg) according to drug type.

mmc1

mmc2
