# Supplementary material for: Investigating the potential impact of dose banding for systemic anti-cancer therapy in the paediatric setting based on pharmacokinetic evidence
Source: Eur J Cancer. 2018 Mar;91:56–67. doi: 10.1016/j.ejca.2017.11.029 (PMC5811050; doi:10.1016/j.ejca.2017.11.029)
Supplement: mmc1 [file mmc1.docx]

**Dosing tables for each drug**

**Table 1S: Dactinomycin 0.5 mg/mL (Additional table not given on NHSE website)**

|  | |  |  |  |
| --- | --- | --- | --- | --- |
| **Band range (mg)** |  | **DOSE GIVEN** | **Variance (percent)** |  |
| **From >=** | **to <** | **Band Dose (mg)** | **Below** | **Above** |
| 0.1595 | 0.1795 | 0.17 | 6 | -6 |
| 0.1795 | 0.1995 | 0.19 | 6 | -5 |
| 0.1995 | 0.22 | 0.21 | 5 | -5 |
| 0.22 | 0.2445 | 0.23 | 4 | -6 |
| 0.2445 | 0.2745 | 0.26 | 6 | -6 |
| 0.2745 | 0.3045 | 0.29 | 5 | -5 |
| 0.3045 | 0.3395 | 0.32 | 5 | -6 |
| 0.3395 | 0.3795 | 0.36 | 6 | -5 |
| 0.3795 | 0.4245 | 0.4 | 5 | -6 |
| 0.4245 | 0.4745 | 0.45 | 6 | -5 |
| 0.4745 | 0.5245 | 0.5 | 5 | -5 |
| 0.5245 | 0.5745 | 0.55 | 5 | -4 |
| 0.5745 | 0.6245 | 0.6 | 4 | -4 |
| 0.6245 | 0.6745 | 0.65 | 4 | -4 |
| 0.6745 | 0.7245 | 0.7 | 4 | -4 |
| 0.7245 | 0.7745 | 0.75 | 3 | -3 |
| 0.7745 | 0.8245 | 0.8 | 3 | -3 |
| 0.8245 | 0.8745 | 0.85 | 3 | -3 |
| 0.8745 | 0.9485 | 0.9 | 3 | -5 |
| 0.9485 | 1.049 | 1 | 5 | -5 |
| 1.049 | 1.149 | 1.1 | 5 | -4 |
| 1.149 | 1.249 | 1.2 | 4 | -4 |
| 1.249 | 1.349 | 1.3 | 4 | -4 |
| 1.349 | 1.449 | 1.4 | 4 | -4 |
| 1.449 | 1.549 | 1.5 | 3 | -3 |
| 1.549 | 1.649 | 1.6 | 3 | -3 |
| 1.649 | 1.7495 | 1.7 | 3 | -3 |
| 1.7495 | 1.8975 | 1.8 | 3 | -5 |
| 1.8975 | 2.0975 | 2 | 5 | -5 |
| 2.0975 | 2.298 | 2.2 | 5 | -4 |
| 2.298 | 2.5455 | 2.4 | 4 | -6 |
| 2.5455 | 2.846 | 2.7 | 6 | -5 |
| 2.846 | 3.1465 | 3 | 5 | -5 |
| 3.1465 | 3.4945 | 3.3 | 5 | -6 |
| 3.4945 | 3.895 | 3.7 | 6 | -5 |
| 3.895 | 4.2955 | 4.1 | 5 | -5 |

**Table 2S: Busulfan 6 mg/mL**

**(Additional part of table, not given on NHSE website, as low doses for children are below doses given in NHS table)**

|  | | | | |
| --- | --- | --- | --- | --- |
| **Band range (mg)** |  | **DOSE GIVEN** | Variance (percent) | |
| **From >=** | **to <** | **Band Dose (mg)** | **Below** | **Above** |
| 1.17 | 1.26 | 1.2 | 3 | -5 |
| 1.26 | 1.38 | 1.32 | 5 | -5 |
| 1.38 | 1.5 | 1.44 | 4 | -4 |
| 1.5 | 1.62 | 1.56 | 4 | -4 |
| 1.62 | 1.74 | 1.68 | 4 | -4 |
| 1.74 | 1.914 | 1.8 | 3 | -6 |
| 1.914 | 2.154 | 2.04 | 6 | -6 |
| 2.154 | 2.394 | 2.28 | 6 | -5 |
| 2.394 | 2.64 | 2.52 | 5 | -5 |
| 2.64 | 2.934 | 2.76 | 4 | -6 |
| 2.934 | 3.294 | 3.12 | 6 | -6 |
| 3.294 | 3.654 | 3.48 | 5 | -5 |
| 3.654 | 4.074 | 3.84 | 5 | -6 |
| 4.074 | 4.554 | 4.32 | 6 | -5 |
| 4.554 | 5.094 | 4.8 | 5 | -6 |
| 5.094 | 5.694 | 5.4 | 6 | -5 |
| 5.694 | 6.294 | 6 | 5 | -5 |
| 6.294 | 6.894 | 6.6 | 5 | -4 |
| 6.894 | 7.494 | 7.2 | 4 | -4 |
| 7.494 | 8.094 | 7.8 | 4 | -4 |
| 8.094 | 8.694 | 8.4 | 4 | -4 |
| 8.694 | 9.294 | 9 | 3 | -3 |
| 9.294 | 9.894 | 9.6 | 3 | -3 |
| 9.894 | 10.494 | 10.2 | 3 | -3 |
| 10.494 | 11.382 | 10.8 | 3 | -5 |
| 11.382 | 12.588 | 12 | 5 | -5 |
| 12.588 | 13.788 | 13.2 | 5 | -4 |
| 13.788 | 14.988 | 14.4 | 4 | -4 |
| 14.988 | 16.188 | 15.6 | 4 | -4 |
| 16.188 | 17.388 | 16.8 | 4 | -4 |
| 17.388 | 18.588 | 18 | 3 | -3 |
| 18.588 | 19.788 | 19.2 | 3 | -3 |
| 19.788 | 20.994 | 20.4 | 3 | -3 |
| 20.994 | 22.77 | 21.6 | 3 | -5 |
| 22.77 | 25.17 | 24 | 5 | -5 |
| 25.17 | 27.576 | 26.4 | 5 | -4 |
| 27.576 | 30.546 | 28.8 | 4 | -6 |
| 30.546 | 34.152 | 32.4 | 6 | -5 |
| 34.152 | 37.758 | 36 | 5 | -5 |

**Table 3S: Carboplatin 10 mg/mL (table given on NHSE website)**

**Table 4S: Cyclophosphamide and Etoposide 20 mg/mL (table given on NHSE website)**
