# Supplementary figures and images for: Investigating the potential impact of dose banding for systemic anti-cancer therapy in the paediatric setting based on pharmacokinetic evidence
Source: Eur J Cancer. 2018 Mar;91:56–67. doi: 10.1016/j.ejca.2017.11.029 (PMC5811050; doi:10.1016/j.ejca.2017.11.029)

**Figure 4SA**


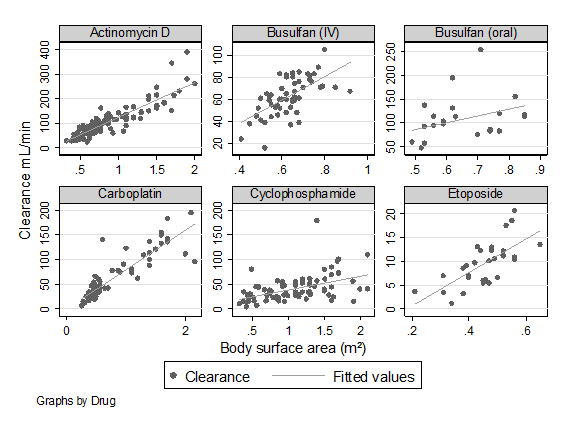


**Figure 4SB**


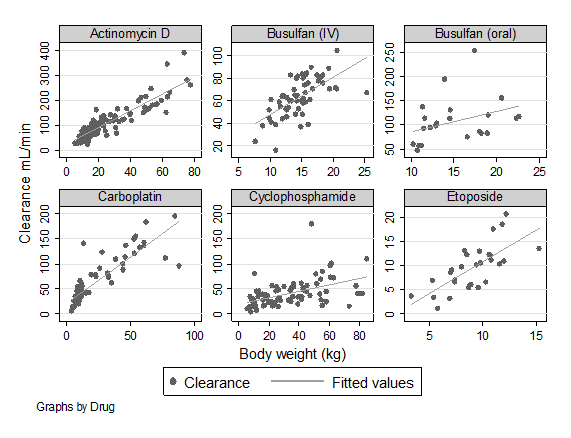

Supplement: mmc2 [file mmc2.docx]
